# The role of bodily experiences during pregnancy on mother and infant outcomes

**DOI:** 10.1111/jnp.12370

**Published:** 2024-05-19

**Authors:** Lydia Beatrice Munns, Catherine Preston

**Affiliations:** ^1^ University of York York UK

**Keywords:** attachment, body satisfaction, interoception, pregnancy

## Abstract

Pregnancy is a transformative time for women and their bodies, and therefore thoughts and feelings and about one's own body and internal bodily sensations may understandably change during this period. Body satisfaction and interoception have been found to influence factors such as antenatal attachment (AA) and maternal mental health. However, mixed results in the literature suggest complex relationships between the bodily experience during pregnancy and outcomes, necessitating a broader investigative approach. We aim to examine the relationship between the pregnancy bodily experience and multiple mother–infant outcomes. It is hypothesised that poor bodily experiences during pregnancy will have negative impacts on these outcomes. Cross‐sectional online survey data was collected from individuals at various gestations throughout pregnancy as part of a larger longitudinal study (*N* = 253, mean age = 32). We analysed validated measures of pregnancy body satisfaction, interoceptive sensibility, AA and mood, as well as intentions to breastfeed. Linear regressions were used to confirm findings from previous literature and a network analysis allowed for a more exploratory approach to understanding the importance of the bodily experience during pregnancy. Multiple regressions found low body satisfaction predicts higher levels of anxiety, depression and AA. A network analysis revealed relationships between body satisfaction and interoception during pregnancy and mother–infant outcomes, including depression and AA. Our results highlight the far‐reaching effects of poor bodily experiences during pregnancy on a variety of outcomes. Understanding the impact of the pregnant bodily experience can help identify at‐risk individuals and inform interventions.

## INTRODUCTION

During pregnancy, women experience multiple changes simultaneously, including rapid hormonal shifts that result in physiological changes such as increased cardiac output (Silversides & Colman, 2008). These changes go hand in hand with changes in physical appearance and body shape, including weight gain, enlarged breasts and swollen ankles, highlighting just how physical the experience of pregnancy is. While pregnancy is often a special time, the pressure to look a certain way – even during pregnancy – is present for many women (Kirk & Preston, 2019).

Body dissatisfaction encompasses negative thoughts and feelings about one's own body (Grogan, 2021). Unfortunately, body dissatisfaction is a pervasive issue for many women, with previous literature suggesting over 20% of females surpass clinical cut offs for weight and shape concerns (Carey et al., 2019). This is an issue given that dissatisfaction with the body is linked to negative effects on well‐being, including disordered eating (Thompson & Stice, 2001), depression (Scheffers et al., 2019), anxiety (Lin & Kulik, 2002), and low self‐esteem (Henriques & Calhoun, 1999), all of which may be particularly damaging during pregnancy.

Pregnancy can shift the focus away from societal pressures to conform to conventional beauty standards, highlighting the significance of reproduction and a new maternal role for women (Davies & Wardle, 1994). However, many of the bodily changes are conflicting with the social ideals of the appearance of the female body, leaving women pressured to maintain a socially desirable pregnant body shape (Hodgkinson et al., 2014). Pregnant women are having to balance the needs of the foetus with societal beauty standards amidst competing sensations, such as increased appetite (Butte & King, 2005) and reduced physical activity due to pain (Vermani et al., 2010) and fatigue (Chien & Ko, 2004). These pressures can impact on how satisfied women are with their changing pregnant bodies (Kirk & Preston, 2019). Previous research has highlighted the complexity of the bodily experience, and demonstrated significant variation in women's attitudes towards the physical changes that occur during pregnancy, with a recent meta‐analysis suggesting some women view the changes as liberating while others find them distressing (Crossland et al., 2023). A recent systematic review explored postnatal bodily experiences and suggested that these were influenced by a variety of personal and societal factors, such as mental health and media influences (Lee et al., 2023). Thus, the authors emphasised the need for a holistic approach to understanding the complexities of women's bodily experiences in the perinatal period, considering individual, social, and institutional factors, to improve antenatal care and policies.

Positive perceptions of the body during pregnancy have been found to help women adapt to the bodily changes (Clark et al., 2009) and have a stronger bond with their unborn child (antenatal attachment; Kirk & Preston, 2019). Low levels of antenatal attachment are associated with fewer positive health and safety practices during pregnancy (Jussila et al., 2020) and less attuned mother–infant interaction after birth (Fuller‐Tyszkiewicz et al., 2012). Further to this, a woman's dissatisfaction with her changing body during pregnancy has been identified as the strongest statistical predictor of antenatal attachment, accounting for up to 27% of the variance alongside depression and relationship satisfaction (Kirk & Preston, 2019). Although this is from cross‐sectional data so cause and effect are only inferred, this may suggest the critical role that the bodily experience during pregnancy can play in maternal and infant well‐being. In contrast, research has also shown negative feelings towards the body to be associated with higher levels of antenatal attachment, suggesting that building a strong bond with the baby and understanding the purpose and function of perinatal bodily changes might help women deal with negative emotions related to their body (Małus et al., 2014). However, these contrasting results might be explained by the lack of pregnancy specific measures in the literature. The lack of specificity could compromise the validity of the collected data and overlook important aspects unique to the pregnancy experience (Fuller‐Tyszkiewicz et al., 2012). This gap in the literature has been addressed by research such as that done by Kirk and Preston (2019), who created the Body Understanding Measure for Pregnancy scale (BUMPs) to measure pregnancy body dissatisfaction.

Poor bodily experiences have also been related to negative experiences for pregnant women and their babies, such as low birth weights (Conti et al., 1998), low rates of breast feeding (Brown et al., 2015) and depression (Schmied & Lupton, 2001). Depression is very prevalent antenatally and postpartum with estimates varying between 5%–25% (Gavin et al., 2005) and 13%–19% respectively (O'Hara & McCabe, 2013). Additionally, experiencing antenatal depression heightens the likelihood of postpartum depression (Yu et al., 2023). Depressive symptoms during pregnancy have been linked to negative experiences for both mothers and babies, including emotional withdrawal, substance abuse and preterm delivery (Bowen & Muhajarine, 2006), as well as reduced breastfeeding (Hamdan & Tamim, 2012) potentially due to the hormonal imbalances associated with depression (O'Hara & McCabe, 2013). A review by Silveira et al. (2015) concluded that not only was there an association between body dissatisfaction and perinatal depression, but that body dissatisfaction could predict both antenatal and postpartum depression. Similarly, anxiety is prevalent during pregnancy, with around 15% of women reporting antenatal anxiety (Rubertsson et al., 2014). Body dissatisfaction has been significantly associated with higher levels of anxiety (Chan et al., 2020; Roomruangwong et al., 2017), which consequently has been associated with lower levels of prenatal attachment (Gioia et al., 2023), and poor psychological well‐being in pregnant women (Allison et al., 2011).

As well as exteroceptive bodily experiences such as body dissatisfaction, internal bodily experiences (interoception) have been associated with antenatal anxiety and depression (Noda et al., 2022; Singh Solorzano et al., 2022; Singh Solorzano & Grano, 2023). Interoception refers to the awareness of, or attention paid to, visceral (internal bodily) signals such as heart rate, hunger, thirst, and pain (Craig, 2002), and can influence how women feel about their bodies, and their babies, during pregnancy. Examining interoception during pregnancy, Clark et al. (2009) gathered qualitative data via interviews, where they reported unique pregnancy experiences that helped pregnant women cope with the bodily changes. For example, tuning into their bodies to feel movement of their baby – presumably due to concern for the needs of the foetus – and demonstrating less avoidance of bodily signals such as pain and discomfort, which has also been found quantitatively (Crossland et al., 2022). Signals from within the body during pregnancy have also been implicated in the parent‐infant relationship, with perceptual experiences such as the baby kicking being associated with stronger levels of foetal attachment (Heidrich & Cranley, 1989) and interoceptive sensibility, an individual's subjective experience of internal bodily sensations, influencing the mother and infant relationship during pregnancy (Stafford et al., 2024) and postpartum (Suga et al., 2022). This highlights that the bodily experience transcends external appearance.

It is therefore unsurprising that a strong connection has been established between body satisfaction and interoception. Notably, Kirk and Preston (2019) found that higher levels of body dissatisfaction during pregnancy are associated with reduced interoceptive sensibility, specifically lower levels of trust in bodily signals (body trusting) and decreased ability to attend to and listen to bodily sensations (body listening). In addition, variations in body dissatisfaction between pregnant and non‐pregnant women have been observed to be fully mediated by their respective levels of body trusting (Crossland et al., 2022). This suggests that interoceptive sensibility could potentially be involved in the relationship between pregnancy and body dissatisfaction.

Together, body dissatisfaction and interoception have been linked to breastfeeding, with research suggesting that women with more concerns around their body appearance are less likely to breastfeed, and of those that do, will breastfeed for a shorter duration compared to those who have fewer concerns around their body (Brown et al., 2015). Concerns among women included fear that breastfeeding would damage their breasts or significantly alter their appearance (Brown et al., 2015). However, breastfeeding offers numerous benefits, including lowering stress levels, thus increasing levels of hormones involved in mother–infant attachment such as oxytocin and prolactin (Jansen et al., 2008). Consequently, decisions regarding breastfeeding play a crucial role in understanding the postnatal bond between mother and baby (Linde et al., 2020). Interoceptive body trusting is one mechanism by which body dissatisfaction could influence breastfeeding. During pregnancy, women commonly experience a sense of losing control over their bodies, with rapid physical changes occurring without conscious effort (Schmied & Lupton, 2001). This can lead to feelings of mistrust in the body, which have the potential to negatively impact the pregnancy and birth experience (Crossland et al., 2022). This holds significant implications for breastfeeding, with qualitative research indicating that when mothers trust in their bodies’ capabilities, it results in a positive and fulfilling breastfeeding experience (Flacking et al., 2021).

Overall, the literature suggests that both body dissatisfaction and interoception could be closely related within the context of perinatal well‐being. The importance of considering these concepts together comes from The Competition of Cues Hypothesis (Pennebaker & Lightner, 1980), suggesting that as attention towards the body is limited, both internal cues (interoception), and external cues (such as body dissatisfaction) are in competition with each other. In line with this hypothesis, it is suggested that body dissatisfaction may divert attention from crucial internal bodily signals which is associated with increased anxiety and depression (Noda et al., 2022; Singh Solorzano et al., 2022; Singh Solorzano & Grano, 2023), and a reduction in antenatal attachment (Heidrich & Cranley, 1989). Despite this, to date no study has comprehensively investigated both interoception and exteroceptive body experiences concurrently in the antenatal period with all these variables. It is hoped that a better understanding of the pregnancy bodily experience will improve awareness surrounding how women feel about their bodies during pregnancy, as currently, a woman's bodily experience is not considered in routine antenatal care settings (Watson et al., 2016). This knowledge could lead to investigations into interventions that target mother's pregnant body dissatisfaction with the aim of improving the pregnancy experience and antenatal well‐being.

### Aims & hypotheses

The present study seeks to use pregnancy specific measures to assess pregnant body dissatisfaction and interoception to identify any potential associations with key mother–infant antenatal variables, including antenatal attachment, anxiety, depression, and breastfeeding intentions. Regressions aim to replicate previous quantitative findings, whereas the primary analysis of a network analysis strives to expand upon this by investigating the relationships among different aspects of the bodily experience and antenatal variables simultaneously. It is predicted that regressions will find higher levels of body dissatisfaction and lower levels of interoception to be associated with lower levels of antenatal attachment, and increased levels of depression and anxiety. As the network analysis is exploratory, beyond the predictions assumed for the regression analyses, no additional predictions are made regarding potential associations. It is expected that there will be a complex display of relationships between body dissatisfaction, interoception, and various mother–infant variables, in the hope that this will shed light on the critical role of the bodily experience in maternal well‐being and the mother–infant relationship during the antenatal period.

## METHODS

### Participants

This research is part of a larger project that aims to investigate the long‐term impact of body dissatisfaction during pregnancy on postnatal variables. The current study received ethical approval from the University of York Departmental Ethics Committee (ref: 122). All participants provided informed consent prior to taking part. Participants responded to online advertisements to take part in an online survey hosted by Qualtrics (https://www.qualtrics.com). The advertisements were distributed via social media sites (Instagram, Facebook), parenting websites (Mumbler, Mumsnet), groups and classes, and local nurseries and GP surgeries.

Overall, 260 pregnant women completed this antenatal questionnaire. See Table 1 for a summary of participant demographics following exclusions. Exclusion criteria included women under 18, those diagnosed with depression or anorexia (self‐disclosed as having a clinical diagnosis assessed via online consent form), incomplete data (those who didn't provide data for any key variables), and past due dates. Two participants were excluded due to incomplete responses and 5 were excluded due to them not being pregnant at the time of completion. This left 253 participants.

**TABLE 1:** Sample demographics.

Demographic	Mean (*SD*)/%
Mean maternal age (*SD*)	31.9 (5.0)
Gender female (%)	100
Mean number of weeks pregnant (*SD*)	27.6 (10.0)
% first pregnancy	55.7
Number of children including pregnancy (*SD*)	1.6 (.8)
Trimester (%)	
First trimester (weeks 1–12)	8.2
Second trimester (weeks 13–26)	38.1
Third trimester (weeks 27–40+)	53.7
Relationship status (%)	
Married/civil partner	58.9
Living with partner	36.8
In a relationship living apart	2.4
Single	2.0
Ethnicity (%)	
White	95.7
Mixed	2.8
Asian	.8
Black, African, Caribbean or Black British	.8
Education level (%)	
School leaver	.4
GCSEs	6.7
NVQ (Level 1–2)	2.0
NVQ (Level 3–5)	8.7
A levels/IB	7.1
HND or BTEC	5.1
Undergraduate degree	37.2
Postgraduate degree	27.7
Doctoral degree	5.1
Employment (%)	
Full time	61.3
Part time	28.5
Student	2.0
Unemployed	8.3
Single births (%)	98.8
Pre pregnancy BMI (*SD*)	25.9 (6.0)
*N = 253*	

### Materials

Qualtrics (Qualtrics, 2020) was used to create and distribute the surveys to participants. Appropriate information, consent and debrief forms were used and presented to participants within the survey.

### Measures

#### Self‐report questionnaires

Pregnant body dissatisfaction, interoceptive sensibility, anxiety, depression, and antenatal attachment were all measured using validated self‐report questionnaires. Please see further details of these in Table 2.

**TABLE 2:** Self‐report questionnaires.

Measure	Items	Subscales	Responses	Scoring	Reliability
Body Understanding Measure for Pregnancy Scale (BUMPs; Kirk & Preston, 2019)	19	*Appearance* (nine items) – dissatisfaction with appearing pregnant; *Weight* (seven items) – concerns about weight gain; *Physical* (three items) – the experience of the physical burdens of pregnancy	Recorded on a 5‐point Likert scale ranging from 1 (*strongly disagree*) to 5 (*strongly agree*)	Sum items for individual subscale scales and all 19 items for a global score. Higher scores indicate more dissatisfaction	Internal consistency (*α* = .71–.91). Test–retest reliability (.78–.93; Kirk & Preston, 2019)
Multi‐dimensional Assessment of Interoceptive Awareness for Pregnancy (MAIA‐Preg; Crossland et al., n.d.)^a^	19	*Emotional Awareness* (4 items) –awareness of bodily signals; *Not‐distracting* (three items) – tendency not to distract from sensations of pain or discomfort; *Attention regulation* (six items) – ability to sustain and control attention to bodily sensations; *Self‐regulation* (three items) – ability to regulate psychological distress by attention to bodily sensations; *Trusting* (three items) – the experience of one's body as safe and trustworthy	Responses are recorded on a 6‐point Likert scale ranging from 0 (*never*) to 5 (*always*)	The score for each scale is calculated by the mean of its individual items. There is no global score	Internal consistency (*α* = .63–.87; Crossland et al., n.d.)
Maternal Antenatal Attachment Scale (MAAS; Condon, 1993)^b^	19	*Quality of Attachment* (10 items) – feelings of closeness and pleasure in interaction; *Strength of Intensity of Preoccupation* (8 items) – extent to which the foetus occupies a central place in the woman's emotional life	Responses are provided on a 5‐point Likert	A Global Attachment Score is calculated from the sum of all 19 items (one item does not load on either subscale)	Reliability (*α* = .82) for the total scale (Condon, 1993)
The Hospital Anxiety and Depression Scale (HADS; Zigmond & Snaith, 1983)	14	*Anxiety* (seven items); *Depression* (seven items)	Statements are rated on a 4‐point Likert scale of 0–3	Subscales are summed to generate an overall subscale score. Higher scores indicate higher anxiety and depression	Internal consistency in pregnant samples (*α* = .74–.81) (Kirk & Preston, 2019)

^a^ The MAIA‐Preg was adapted from the Multi‐dimensional Assessment of Interoceptive Awareness scale (MAIA; Mehling et al., 2012). Questions that were not pertinent to pregnant women were removed, and the subscales underwent refinement through a factor analysis. Consequently, the MAIA subscales of ‘not worrying’, ‘body listening’ and ‘noticing’ were eliminated from the scale.

^b^ For ethical reasons the current study omitted two items contributing to the intensity scale relating to feelings of wanting to punish the foetus and feelings if the pregnancy were to be lost.

#### Feeding intentions

Feeding intentions were gathered by asking participants how they intended to feed their baby (with response options including breastfeeding, formula or combination feeding). Similar questions on breastfeeding have been included in previous research (Brown et al., 2015). Those who did not select breastfeeding were coded as 0. If breastfeeding was selected, a follow up question was presented asking how long women intended to breastfeed for. Responses were standardised on a Likert scale consisting of six responses coded 1 (less than 1 month), 2 (between 1 and 3 months), 3 (between 3 and 6 months), 4 (between 6 and 9 months), 5 (between 9 and 12 months) and 6 (more than 12 months).

### Procedure

#### Survey

Participants were invited to complete the surveys online via a Qualtrics link. They were presented with an information sheet and provided with tick boxes to consent to taking part. They were then presented with the questions, followed by a debrief form.

### Data analysis

#### Regression analyses

Global and subscale scores were appropriately calculated for all the measures according to the relevant scoring guidelines. Data was checked for assumptions for a linear regression, including normality, multicollinearity, linearity, and homoscedasticity. All possible theory‐based covariates were included in initial regression models, with those not showing statistical significance (>.05) being removed to maintain model parsimony. Possible covariates included gestation (weeks pregnant), age, parity, BMI, education level and relationship status.

Linear regressions were used to assess the relationship between the regressors (body dissatisfaction and interoceptive body trusting) and antenatal variables, including attachment, anxiety, and depression, in order to directly replicate previous findings. Due to the lack of a comprehensive global interoception score in the MAIA‐Preg, the ‘body trusting’ subscale was used. This decision was informed by its established association with body dissatisfaction among pregnant women (Crossland et al., 2022) and antenatal attachment (Stafford et al., 2024). Further to this, its consistency as a subscale in both the MAIA and MAIA‐Preg frameworks (Crossland et al., n.d.) demonstrates its reliability and relevance in this context. Single forced entry regressions were used as covariates included were considered as potential bias variables, rather than additional regressors. Analyses were conducted using Python Jupyter Lab (Kluyver et al., 2016) using the OLS module from the statsmodels package (Perktold et al., 2023).

#### Partial correlation network analyses

A partial correlation network analysis was conducted to explore the relationships between all the data gathered during the antenatal period including body dissatisfaction, antenatal attachment, interoceptive sensibility, anxiety, depression and feeding intentions. This was done using a partial correlation matrix using the q‐graph package in RStudio (Epskamp et al., 2012; RStudio Team, 2020). A mixed graphical model using partial correlation was used instead of Gaussian models due to the lack of multivariate normality across the data (*p* = .025) and due to the inclusion of categorical and continuous data. Regularisation was not implemented to the network due to the violation of the assumption of sparsity. Although the network is exploratory, all included nodes are predicted to play a role in the network. Thus, implementing regularisation, which imposes sparsity on the network, may miss some meaningful associations between nodes of interest. The network was bootstrapped 1000 times using the Bootnet package in RStudio, allowing us to calculate confidence intervals for each of the partial correlations or edge weights in the network. To assess the stability of the network, we conducted case‐dropping bootstrapping using the Bootnet package in RStudio (Epskamp et al., 2018), enabling us to evaluate the robustness of the network by systematically removing cases and analysing its impact on the results. Due to the exploratory nature of this analysis, potential covariates were added as nodes due to their relationship with body dissatisfaction in previous literature, including age (Tiggemann & McCourt, 2013), gestation (Skouteris et al., 2005) and BMI (Quittkat et al., 2019).

## RESULTS

### Descriptive statistics

Overall, 253 participants were included in analyses. See Table 3 for detailed descriptives.

**TABLE 3:** Descriptive statistics of independent and dependent variables.

Measure	Mean (*SD*)	Range
BUMPS (*SD*)^a^	59.7 (13.6)	33–95
BUMPS appearance	26.4 (6.8)	9–45
BUMPS physical	11.5 (3.1)	3–15
BUMPS weight	21.8 (6.8)	7–35
AA (*SD*)^b^	66.6 (7.2)	45–81
AA intensity	27.5 (4.4)	14–38
AA quality	34.6 (3.5)	22–40
MAIA_Preg (*SD*)^c^		
MAIA_Preg emotional awareness	11.9 (4.2)	0–20
MAIA_Preg not distracting	6.0 (2.9)	0–14
MAIA_Preg attention regulation	15.7 (5.3)	0–30
MAIA_Preg self‐regulation	8.0 (3.2)	0–15
MAIA_Preg trusting	8.6 (3.7)	0–15
HADS (*SD*)^b^		
Anxiety	8.0 (3.7)	1–18
Depression	5.7 (3.1)	0–17
Feeding intention (%)^a^		
Breastfeeding	68.4	
Formula feeding	20.5	
Combination feeding	11.1	
Intended breastfeeding duration (%)^d^		
Less than 1 month	25.3	
Between 1 and 3 months	24.9	
Between 3 and 6 months	22.7	
Between 6 and 9 months	16.0	
Between 9 and 12 months	7.6	
More than 12 months	3.6	

^a^ *N* = 253.

^b^ *N* = 201.

^c^ *N* = 197.

^d^ *N* = 225.

### Linear regressions

**FIGURE 1:**
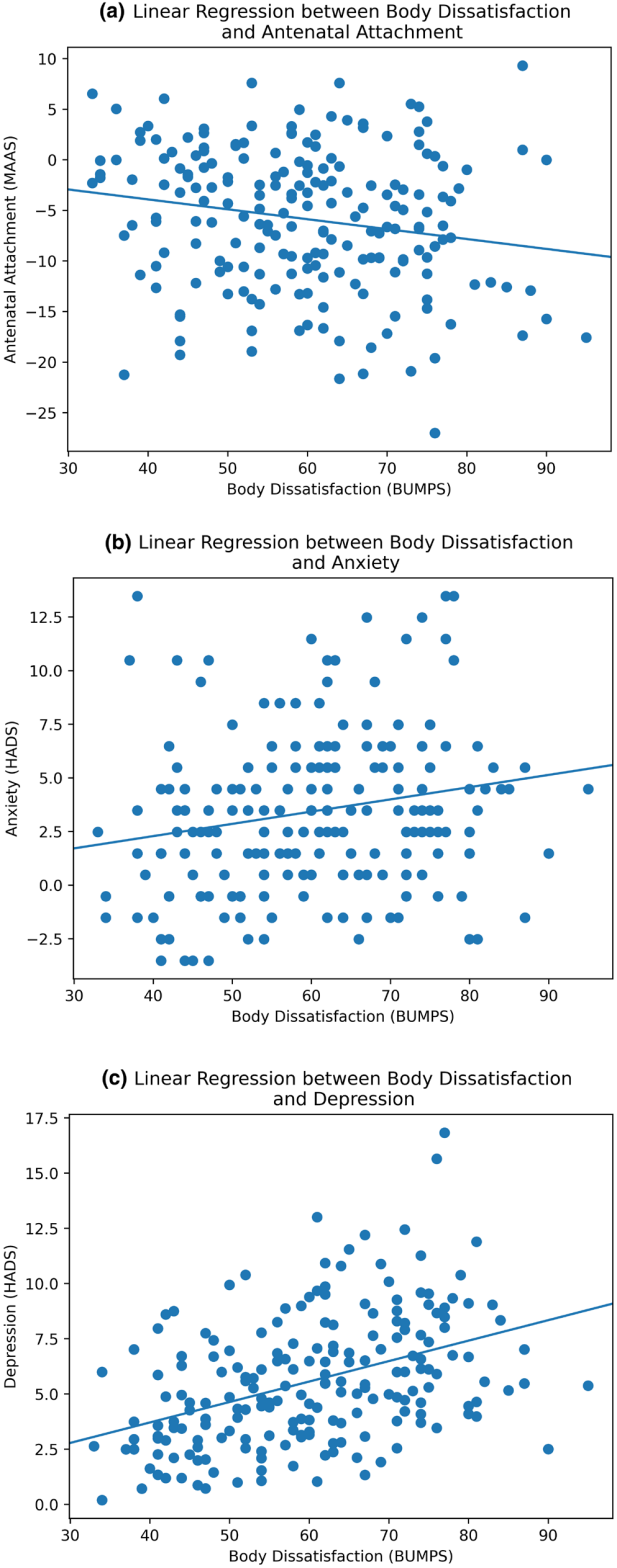
Regression plots showing the relationship between body dissatisfaction and antenatal attachment, anxiety, and depression (from right to left).

**FIGURE 2:**
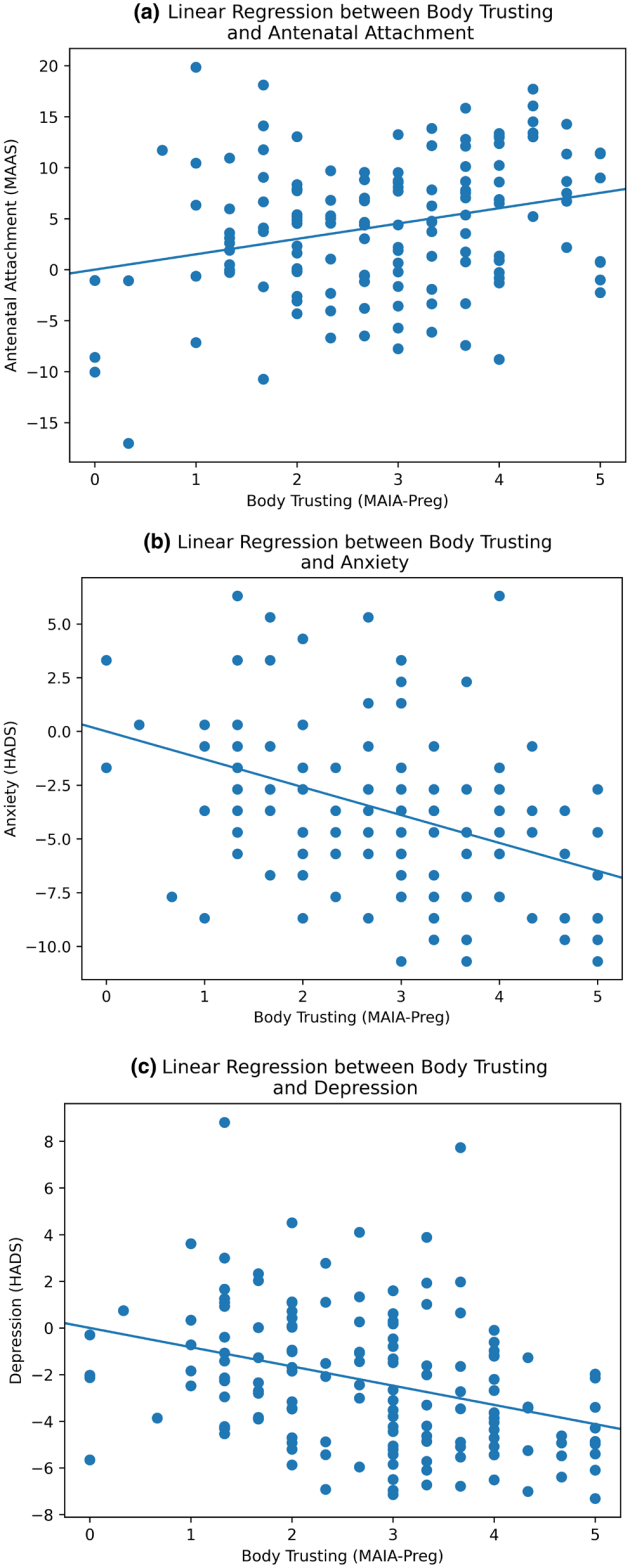
Regression plots showing the relationship between interoceptive body trusting and antenatal attachment, anxiety, and depression (from right to left).

We conducted linear regression analyses to examine the relationship between the regressors (body dissatisfaction and interoceptive body trusting) and multiple variables including antenatal attachment, anxiety, and depression, in order to replicate previous findings. See Table 4 for results considering covariates and Figures 1 and 2 for visualisations of the variables of interest. For regression results without covariates, see Appendix S1A.

**TABLE 4:** Regression results with body dissatisfaction and body trusting as regressors.

	df	Beta	*t* value	*p* value	*F*	*F p* value	Adjusted *R* ^2^
Body dissatisfaction							
Antenatal attachment^a^ ^,^ ^b^	197	−.098	−2.61	.010	7.81	<.001	.093
Anxiety^d^	199	.057	2.92	.004	8.51	.004	.036
Depression^a^ ^,^ ^c^	197	.093	6.27	<.001	18.85	<.001	.211
Body trusting							
Antenatal attachment^a^ ^,^ ^b^	140	1.506	3.26	.001	9.56	<.001	.152
Anxiety^d^	145	−1.297	−5.47	<.001	29.88	<.001	.165
Depression^a^ ^,^ ^c^	143	−.825	−4.22	<.001	10.02	<.001	.156

*Note*: Distinct regression models were employed for each variable. Beta coefficients pertain specifically to the relationship between the body dissatisfaction or interoceptive body trusting and the mentioned variable.

^a^ Parity as a covariate.

^b^ Gestation as a covariate.

^c^ Age as a covariate.

^d^ No covariates.

### Partial correlation network analysis

**FIGURE 3:**
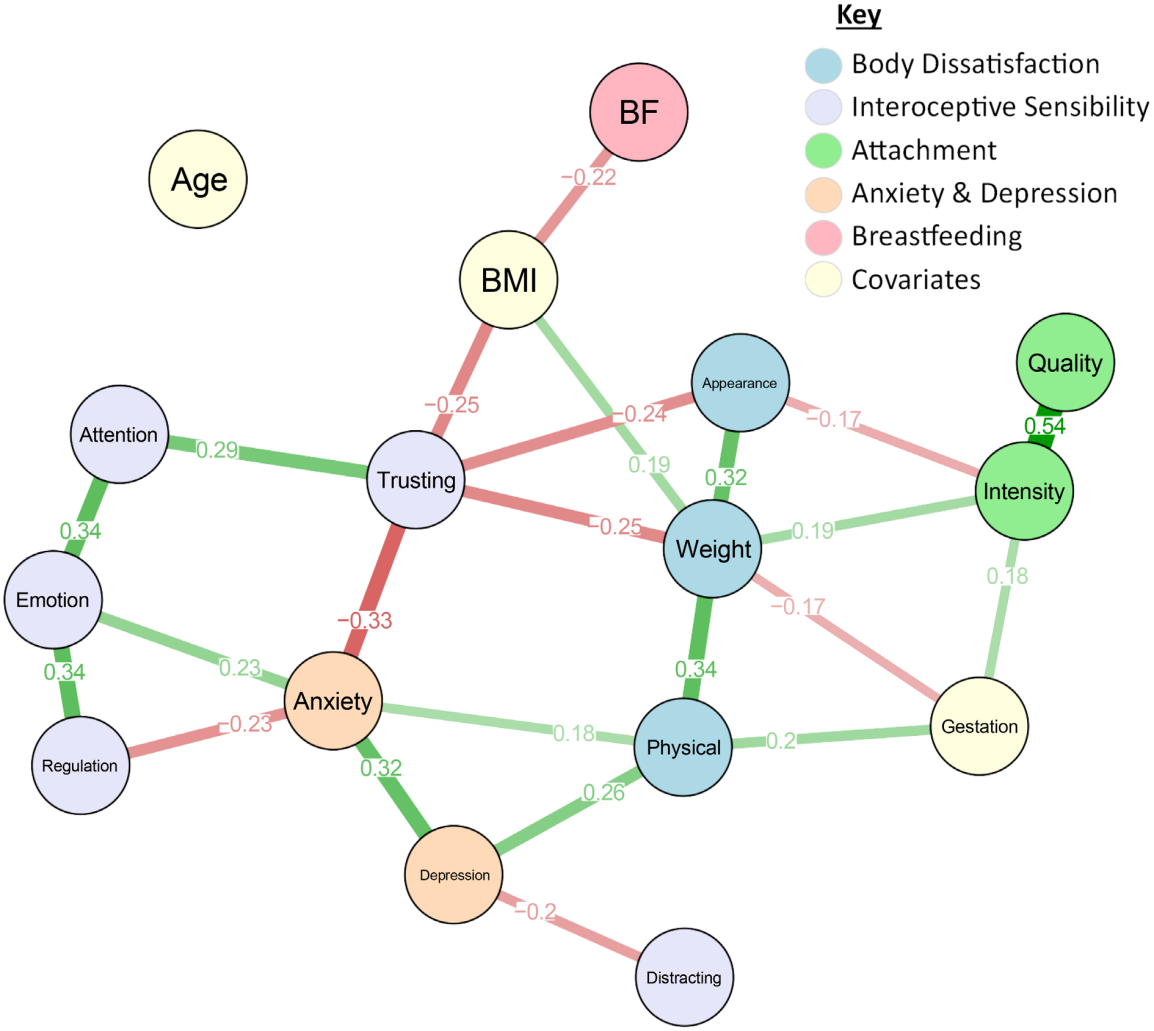
A partial correlation network analysis to show the relationships between body dissatisfaction during pregnancy and mother–infant variables. Nodes represent variables, and edges represent the strength of the connections between nodes. Green edges represent positive relationships and red edges represent negative relationships.

The network in Figure 3 examines the relationships between the BUMPS subscales and multiple variables. The network consisted of 16 nodes representing individual variables and their connections. The decision was made to include the subscales of all the measures collected, particularly those of the BUMPS, in order to further delve into the relationships outlined by the regression analyses above. Resample and case‐drop bootstrapping were applied, showing a robust and stable network. Please see further details in Appendix S1B.

Four key network properties were investigated: edge weights, degree centrality, strength centrality, betweenness centrality and clustering.

#### Edge weights

Edge weights are the values assigned to the connections or ‘edges’ within a network. The edge density (ratio between the actual and possible number of edges) was calculated as .192, suggesting a relatively sparse network.

The edge weights show significant negative relationships between the appearance BUMPS subscale and antenatal attachment intensity and interoception (body trusting), suggesting that higher dissatisfaction with the body's appearance is related to lower levels of attachment and body trust. The weight BUMPS subscale was positively associated with antenatal attachment intensity and negatively associated with body trusting, suggesting that higher dissatisfaction with the body's weight is related to higher levels of attachment and lower levels of body trust. The physical BUMPS subscale showed positive relationships with depression, gestation and anxiety, suggesting that higher dissatisfaction with the body's physical ability is associated with higher levels of depression, anxiety, and a later gestation.

Interoceptive subscales were found to be associated with anxiety and depression, but none directly with antenatal attachment. Specifically, higher levels of anxiety were linked with lower levels of interoceptive self‐regulation and body trusting, and higher levels of interoceptive emotional awareness. Higher levels of depression were associated with the interoceptive not‐distracting subscale.

#### Centrality

Analysis into the degree centrality of nodes (how well connected one node is to other nodes), found that nodes varied in their number of connections, ranging from zero to six. The BUMPS weight subscale and body trusting nodes had the highest degree centrality, with six and five connections respectively.

Strength centrality (how well connected one node is to other nodes, considering the weights associated with those connections), suggests that the BUMPS weight subscale was the strongest (1.46), closely followed by body trusting (1.35).

Betweenness centrality shows the extent to which a certain node lies on the shortest paths between other nodes, with high betweenness centrality indicating that the node acts as a ‘bridge’ to other nodes, encouraging the connections within the network. Our results suggest that body trusting was the node with the highest betweenness centrality (35), followed closely by the BUMPS weight subscale (32). See Appendix S1C for all node centrality coefficients.

#### Clustering

The node clustering coefficient measures the extent to which the neighbours of a specific node are connected to each other. Nodes with the highest clustering coefficient indicate a large density of connections around them. Interoceptive self‐regulation had the highest clustering coefficient of 1, suggesting that all its neighbouring nodes were directly connected to each other. This node was followed by the BUMPS appearance subscale and gestation, both with clustering coefficients of .67. This suggests that these variables have a relatively high local clustering pattern within the network, and a significant proportion of the nodes they connected with connect with each other. Nodes exhibiting higher clustering coefficients may assume a less pivotal role within the broader network, as their neighbouring nodes are interconnected. Consequently, these nodes can be viewed as contributing less unique or distinctive information to the network (Costantini & Perugini, 2014). See Appendix S1C for all node clustering coefficients.

## DISCUSSION

The current findings reveal associative relationships between aspects of the bodily experience, specifically body dissatisfaction and interoceptive body trusting, and antenatal factors, such as antenatal attachment, anxiety and depression. These associations suggest that the way women feel about their bodies and internal signals during pregnancy relates to aspects of their mental well‐being, and their relationship with their unborn baby. A clearer understanding of these relationships has provided insight into the importance of the bodily experience in mother–infant well‐being and highlights a rationale for more in‐depth research into the mechanisms behind these relationships.

Examining the regressions reveals that lower levels of body dissatisfaction and higher interoceptive body trusting are associated with higher levels of antenatal attachment, and lower levels of anxiety and depression. This is in agreement with the previous literature (Stafford et al., 2024; Kirk & Preston, 2019) and supports our hypotheses, confirming the importance of the bodily experience during pregnancy. The amount of variance explained by the bodily experience was particularly interesting, with body dissatisfaction accounting for more variance in depression scores compared to anxiety and attachment. Body dissatisfaction during pregnancy has been found to be more strongly associated with depression than anxiety (Kirk & Preston, 2019), likely attributed to the physiological demands placed upon the body, consequently limiting women from engaging in activities they previously enjoyed prior to pregnancy. In comparison, interoceptive body trusting accounted for more consistent variance across attachment, anxiety and depression, potentially suggesting a more fundamental role in mother and infant factors. While the regression analyses offered valuable insights into the overarching connection between the bodily experience and mother–infant variables, there was a lack of depth surrounding the intricate interactions among subscales within the broader constructs.

The holistic approach of the network analysis shed light on this matter by revealing the facets of body dissatisfaction and interoception that exhibited stronger connections with specific variables of interest. Notably, dissatisfaction regarding body appearance, weight and physical ability were distinctively linked to maternal and infant well‐being. Concern with body weight and appearance were associated with lower body trusting, thus being dissatisfied with the body may lead to less trust in bodily sensations, or vice versa. Research has already suggested that body dissatisfaction and interoception are inherently linked both in pregnant and non‐pregnant samples (Crossland et al., 2022), and that body weight concerns may play a key role in interoceptive sensations during pregnancy (Kirk & Preston, 2019). It is therefore understandable that the network implicates body mass index (BMI) in the relationship between body dissatisfaction and interoception, with women who reported higher BMIs before pregnancy reporting increased levels of weight dissatisfaction and body trusting. This is consistent with previous literature, with high BMI being associated with poorer interoception (Robinson et al., 2021) and body dissatisfaction (Quittkat et al., 2019).

As pregnancy progresses, body weight typically increases due to the growing foetus. The network suggests dissatisfaction with body weight tends to decrease as gestation increases supporting previous literature (Crossland et al., 2022), however contrary to the link between weight dissatisfaction and pre‐pregnancy BMI. Skouteris et al. (2005) found that women in their 3rd trimester felt less fat compared to those earlier in pregnancy, suggesting that this may be due to looking more noticeably pregnant at later gestation. This could explain why individuals in the current study experienced fewer weight concerns as pregnancy progressed, suggesting a distinct disparity between how body dissatisfaction is viewed between pregnant and non‐pregnant bodies. This disparity further supports the use of pregnancy specific measures to capture the bodily experience which were used in the current research via the Body Understanding Measure for Pregnancy Scale (Kirk & Preston, 2019) and the Multidimensional Assessment of Interoceptive Awareness for Pregnancy (Crossland et al., n.d.). Measures for use among the general population may fail to capture nuanced aspects of the bodily experience during pregnancy, and so pregnancy specific measures may make data more relevant to the unique bodily changes and experiences of pregnancy, thus enhancing the accuracy and validity of findings.

The network was able to further clarify the involvement of intentions to breastfeed within the wider context of the bodily experience, revealing that breastfeeding intention was indirectly related to weight and physical dissatisfaction and interoceptive body trusting, through BMI. Therefore, those with higher BMI's had less intention to breastfeed, and for those that did intend to breastfeed, this was for a shorter period. This aligns with previous literature which has linked both body dissatisfaction (Brown et al., 2015) and BMI (Amir & Donath, 2007) with breastfeeding intentions. It has been suggested that this could be due to socio‐cultural factors, with obese women being more likely to come from lower socio‐economic backgrounds and engage in fewer health and safety practices during pregnancy (Amir & Donath, 2007), as well as cultural factors, such as embarrassment of breastfeeding in public and concerns around the impact of breastfeeding on the appearance of breasts (Brown et al., 2015). Although these results were consistent with our hypotheses, the current sample were self‐selecting, majority white and well‐educated, with 79.5% intending to breastfeed. Therefore, firm conclusions cannot be drawn from such a limited sample. Further research is also needed to uncover what happens postnatally, as some mothers may change their mind, never start breastfeeding or breastfeed for a shorter or longer period than they initially planned.

Interestingly, not all the relationships displayed within the network were consistent with our predictions, for example, high levels of dissatisfaction with body weight was associated with higher levels of antenatal attachment intensity (frequency of thoughts about the baby), but not attachment quality (affective valance of the thoughts about baby). Małus et al. (2014) studied the relationship between body dissatisfaction during pregnancy and antenatal attachment by gathering self‐report data from 100 women in their 2nd Trimester. Their analysis showed that greater dissatisfaction with the body during pregnancy was related to stronger attachments, concluding that despite body dissatisfaction, it is still possible to develop a positive bond with the foetus. One explanation of this outcome could stem from the idea that women who are more dissatisfied with their body weight are focusing on their body more which could lead to them thinking more about the baby (attachment intensity) but not necessarily thinking more negatively about the baby (attachment quality). Therefore women who spend more time thinking about their bodies may be more likely to have a stronger relationship with their babies (Kirk & Preston, 2019). Alternatively, focusing on the baby's well‐being and anticipating the joy of motherhood could help alleviate any negative feelings about their weight, and serve as an emotional coping mechanism. However, the relationship between body weight dissatisfaction and antenatal attachment is in contrast with the networks finding that dissatisfaction with body appearance was associated with lower attachment intensity, supporting the majority of previous literature looking at body satisfaction and antenatal attachment (e.g. Kirk & Preston, 2019). Appearance and weight related body dissatisfaction subscales may have different effects since weight gain may be more directly related to physical changes and health of the unborn baby (e.g. ’I feel like my bump is too big’), compared to general body appearance (e.g. ‘It upsets me when people comment on my changing body’). A third variable that could be playing an important role here is parity, with body dissatisfaction being elevated (Crossland et al., 2022) and antenatal attachment being lower (Condon & Esuvaranathan, 1990) among those who already have children, suggesting this should be considered in further analyses.

Overall, the network implicates the bodily experience in attachment intensity rather than quality. This suggests that thoughts and feelings towards the body are more strongly associated with how often women think about the baby rather than whether those thoughts are positive or negative. Incorporating the competition of cues hypothesis (Pennebaker & Lightner, 1980) could shed light on this, as it suggests individuals who focus more on external cues, such as societal standards of body image during pregnancy, may pay less attention to signals from within the body, which as well as interoception, could also incorporate sensations directly from the foetus. Future research should gain a more comprehensive understanding of whether metrics assessing bodily experiences encompass concepts that impact the mental capacity to think about the foetus. This exploration could reveal whether dissatisfaction with appearance potentially diminishes this cognitive capacity, while a strong sense of body trusting might enhance it by preventing excessive worry and hypervigilance.

As well as revealing how body weight and appearance dissatisfaction relate to the mother–infant relationship, the network highlights the significance of dissatisfaction with the body's physical abilities in maternal mental well‐being, supporting existing research findings (e.g. Kirk & Preston, 2019). Changes during pregnancy can result in women being unable to do things that they had done pre‐pregnancy potentially due to fatigue (Chien & Ko, 2004) and pain (Vermani et al., 2010). Dissatisfaction around the bodies’ physical abilities could contribute to feelings of depression and anxiety due to a reduction in endorphin release, socialising, and stress relief activities (Marín‐Jiménez et al., 2022). Interoception also seems to play an important role in levels of anxiety and depression, with low levels of trusting and self‐regulatory interoception, and high emotional awareness being associated with increased anxiety symptoms. Previous literature has implicated interoception in anxiety, with anxious individuals more likely to pay attention to internal bodily signals (as a way of protecting themselves from danger), and less able to self‐regulate interoceptive signals and trust the body (Garfinkel et al., 2015; Hsueh et al., 2023).

Looking more holistically at the network, the centrality of nodes suggests that body weight dissatisfaction and interoceptive body trusting were the most significant nodes in the network. Therefore, these nodes play a crucial role in mediating the connections and information flow within the network, exerting a substantial influence on other variables, and contributing significantly to the overall network dynamics and interactions. The network showed a high clustering coefficient for the node of interoceptive self‐regulation, suggesting its neighbouring nodes – interoceptive emotional awareness and anxiety – are highly interconnected. While this supports the established link between interoception and anxiety (Garfinkel et al., 2015), it also implies that interoceptive regulation's role in anxiety is not unique, underscoring the importance of considering interoception's multifaceted nature and its varied contributions to anxiety across different contexts (Costantini & Perugini, 2014). The use of a partial correlation network analysis, a novel statistical technique in this research area, has allowed for a comprehensive exploration of the interconnectedness between body dissatisfaction, interoception and various mother and infant variables. The network analysis technique captures the holistic nature of the bodily experience during pregnancy and goes beyond simple associations. By examining the frequency, strength, and direction of connections between variables, while accounting for other variables in the network, the analysis provides a nuanced understanding of their complex relationships and highlights the importance of both body dissatisfaction and interoception in the wider context of mother–infant well‐being.

The inherent connection between body dissatisfaction and interoception, as observed in our pregnancy network analysis, holds implications not only for the well‐being of expectant mothers but also for broader contexts of general well‐being and other critical life stages characterised by significant physical transformations, such as menopause and puberty. This model, although specific to pregnancy, can serve as a lens through which we can examine the complex associations between body dissatisfaction and mental health and well‐being. The significant bodily changes experienced during pregnancy make it an ideal focal point for investigating these dynamics. Consequently, heightened interoceptive attention could potentially impact the relationships between body dissatisfaction and well‐being.

### Limitations

As is the nature of research, there were limitations to the methodology of the current study. Firstly, the use of self‐report measures relies on the honesty and accuracy of participant responses, with participants potentially providing socially desirable answers. This is a particular issue when gathering data on levels of antenatal attachment, as negative feelings towards their unborn babies is something many women feel uncomfortable disclosing, partly due to the social stigma (Filippetti et al., 2022). Further to this, the self‐report format may result in sampling bias, as it may attract a certain population who have the time to complete the research and find the research easily accessible. This can be seen in the current sample, with 70% being educated to degree level of above.

Another issue is the lack of ethnic diversity among the participants, with most participants taking part being from a white background. Lack of representation of ethnic minority groups is a wide‐reaching issue across health care research (Redwood & Gill, 2013), which may partly contribute to poorer experiences within maternity settings for non‐white individuals (Henderson et al., 2013). Previous literature has suggested differences between ethnicities when it comes to body dissatisfaction among the general population (Miller et al., 2000) and further research is needed to better understand the differences in body dissatisfaction during pregnancy between different ethnic groups.

Finally, due to the nature of the analyses conducted, and the cross‐sectional design, causality and directionality cannot be established, so we cannot definitively confirm whether poorer variable scores are partly caused by poorer bodily experiences, or vice versa. We also chose not to employ statistical regularisation (to protect against overfitting) in our network analysis, as detailed in the methods section. While this decision aligns with our assumptions and objectives, it is essential to recognise it as a potential limitation, as it may lead to the exclusion of certain less robust associations between variables. However, the edge density and bootstrapping statistics suggest that overfitting is unlikely to be an issue in the current network.

### Implications & conclusions

These findings highlight the importance of integrating discussions on the bodily experience into antenatal health care appointments, ensuring that the emotional and psychological aspects of pregnancy are also considered. This could also contribute to reducing the stigma around discussing body dissatisfaction during pregnancy, with the physical health of both mother and baby often prioritised over maternal mental well‐being (Hodgkinson et al., 2014). Furthermore, this research suggests a need for interventions aimed at improving the bodily experience during pregnancy. For example, yoga has been found improve well‐being through improving mindfulness, feelings towards the body and interoceptive awareness (Tihany et al., 2016), and has shown promise for improving maternal well‐being both antenatally (Jiang et al., 2015) and postnatally (Munns et al., 2024).

To conclude, the results clearly illustrate that the bodily experience is multifaceted and significantly relates to various factors, with dissatisfaction with pregnant body weight and interoceptive body trusting being strongly associated with maternal and infant well‐being. Looking at body dissatisfaction in a one dimensional or simplistic way may limit our understanding of the nuanced connections between body dissatisfaction and maternal and infant well‐being. The network analysis shows us how central these concepts are to our understanding of the wider context regarding mother and infant well‐being. Future research needs to investigate the long‐term impacts of the perinatal bodily experience and how interventions promoting the bodily experience during pregnancy could improve the well‐being of mother and baby.

## AUTHOR CONTRIBUTIONS

**Lydia Beatrice Munns:** Conceptualization; investigation; writing – original draft; funding acquisition; methodology; validation; writing – review and editing; project administration; formal analysis; visualization; data curation. **Catherine Preston:** Conceptualization; investigation; methodology; validation; writing – review and editing; supervision; formal analysis; data curation; visualization; funding acquisition; project administration.

## CONFLICT OF INTEREST STATEMENT

None.

## Supporting information


Appendix S1.

## Data Availability

The data that support the findings of this study are openly available in OSF at https://osf.io/57kc2/ DOI 10.17605/OSF.IO/57KC2.
