# Supplementary material for: The role of bodily experiences during pregnancy on mother and infant outcomes
Source: J Neuropsychol. 2024 May 19;19(Suppl 1):131–51. doi: 10.1111/jnp.12370 (PMC11923730; doi:10.1111/jnp.12370)
Supplement: Supplementary file 1 — Appendix S1. [file JNP-19-131-s001.docx]

**Supplementary Materials**

**A**

***Table A.*** *Regression results without covariates. Body dissatisfaction and body trusting as regressors.*

|  | **df** | | **Beta** | | **t value** | | ***p* value** | | **F** | | **F *p* value** | | **Adjusted R^2^** | |
| --- | --- | --- | --- | --- | --- | --- | --- | --- | --- | --- | --- | --- | --- | --- |
| **Body dissatisfaction** |  | |  | |  | |  | |  | |  | |  | |
| Antenatal attachment | | 199 | -0.121 | | -3.25 | | 0.001 | | 10.56 | | 0.001 | | 0.046 | |
| Anxiety | | 199 | 0.057 | | 2.92 | | 0.004 | | 8.51 | | 0.004 | | 0.036 | |
| Depression | | 199 | 0.098 | | 6.56 | | <0.001 | | 42.98 | | <0.001 | | 0.173 | |
| **Body trusting** | |  |  | |  | |  | |  | |  | |  | |
| Antenatal attachment | | 142 | 1.553 | | 3.30 | | 0.001 | | 10.90 | | 0.001 | | 0.065 | |
| Anxiety | | 145 | -1.297 | | -5.47 | | <0.001 | | 29.88 | | <0.001 | | 0.165 | |
| Depression | | 145 | -0.873 | | -4.42 | | <0.001 | | 19.51 | | <0.001 | | 0.112 | |
| *Distinct regression models were employed for each variable. Beta coefficients pertain specifically to the relationship between the body dissatisfaction or interoceptive body trusting and the mentioned variable.* | | | | | | | | | | | | | | |

**B**

**Network Analysis Bootstrapping**

We employed bootstrapping with 1000 resamples to enhance the robustness of our network analysis. Table B shows the results, including edge weights and corresponding confidence intervals (CIs) for the bootstrapped network.

We further assessed the stability of the relationships between variables using case-dropping bootstrapping. To maintain a correlation of 0.7 or higher in at least 95% of the samples, we found that a maximum of 51.8% of cases could be dropped without significantly affecting the network edges, and up to 28.5% of cases could be dropped while retaining the strength of associations. These results show the robustness of our network and provide valuable guidance for data inclusion and analysis.

***Table B.*** *Bootstrapping results showing edge weights and their confidence intervals.*

| **Nodes** | **Edge Weight** | **Lower CI** | **Upper CI** |
| --- | --- | --- | --- |
| AA Intensity — Gestation | 0.18 | -0.01 | 0.36 |
| AA Quality — AA Intensity | 0.54 | 0.41 | 0.65 |
| Breastfeeding — BMI | -0.22 | -0.36 | -0.04 |
| BUMPs Physical — BUMPs Weight | 0.34 | 0.21 | 0.47 |
| BUMPs Physical — HADS Anxiety | 0.18 | 0.00 | 0.32 |
| BUMPs Physical — HADS Depression | 0.26 | 0.08 | 0.39 |
| BUMPs Physical — Gestation | 0.20 | 0.07 | 0.33 |
| BUMPs Appearance — AA Intensity | -0.17 | -0.35 | 0.02 |
| BUMPs Appearance — MAIAP Trusting | -0.24 | -0.39 | -0.07 |
| BUMPs Weight — AA Intensity | 0.19 | 0.02 | 0.35 |
| BUMPs Weight — BMI | 0.19 | 0.02 | 0.35 |
| BUMPs Weight — BUMPs Appearance | 0.32 | 0.18 | 0.46 |
| BUMPs Weight — MAIA-Preg Trusting | -0.25 | -0.40 | -0.10 |
| BUMPs Weight — Gestation | -0.17 | -0.30 | -0.03 |
| HADS Anxiety — HADS Depression | 0.32 | 0.16 | 0.50 |
| HADS Anxiety — MAIA-Preg Awareness | 0.23 | 0.02 | 0.46 |
| HADS Anxiety — MAIA-Preg Regulation | -0.23 | -0.42 | -0.04 |
| HADS Anxiety — MAIA-Preg Trusting | -0.33 | -0.52 | -0.15 |
| HADS Depression — MAIA-Preg Distracting | -0.20 | -0.42 | -0.03 |
| MAIA-Preg Attention — MAIA-Preg Trusting | 0.29 | 0.13 | 0.44 |
| MAIA-Preg Awareness — MAIA-Preg Attention | 0.34 | 0.19 | 0.50 |
| MAIA-Preg Awareness — MAIA-Preg Regulation | 0.34 | 0.20 | 0.49 |
| MAIA-Preg Trusting — BMI | -0.25 | -0.45 | -0.08 |
| *CI = Confidence Interval, MAIA-Preg = Multidimensional Assessment of Interoceptive Awareness for Pregnancy, BUMPs = Body Understanding Measure for Pregnancy, HADS = Hospital Anxiety and Depression Scale* | | | |

**C**

| **Node** | **Strength Centrality** | **Betweenness Centrality** | **Clustering** |
| --- | --- | --- | --- |
| BUMPS Physical | 0.976 | 13 | 0.333 |
| BUMPS Weight | 1.461 | 32 | 0.333 |
| BUMPS Appearance | 0.728 | 0 | 0.666 |
| Anxiety | 1.285 | 23 | 0.200 |
| Depression | 0.781 | 13 | 0.333 |
| Attachment Quality | 0.535 | 0 | 0.000 |
| Attachment Intensity | 1.078 | 13 | 0.333 |
| Emotional Awareness | 0.909 | 1 | 0.333 |
| Not-Distracting | 0.201 | 0 | 0.000 |
| Attention Regulation | 0.625 | 7 | 0.000 |
| Self-Regulation | 0.572 | 0 | 1.000 |
| Body Trusting | 1.349 | 35 | 0.200 |
| Breastfeeding Intention | 0.221 | 0 | 0.000 |
| Age | 0.000 | 0 | 0.000 |
| Gestation | 0.548 | 0 | 0.666 |
| BMI | 0.668 | 13 | 0.333 |

***Table C.*** *Network centrality and clustering coefficients*
